# Sparse Auto-Calibration for Radar Coincidence Imaging with Gain-Phase Errors

**DOI:** 10.3390/s151127611

**Published:** 2015-10-30

**Authors:** Xiaoli Zhou, Hongqiang Wang, Yongqiang Cheng, Yuliang Qin

**Affiliations:** School of Electronic Science and Engineering, National University of Defense Technology, Changsha 410073, China; E-Mails: oliverwhq1970@gmail.com (H.W.); nudtyqcheng@gmail.com (Y.C.); yuliang.qin@gmail.com (Y.Q.)

**Keywords:** radar coincidence imaging (RCI), sparse recovery, orthogonal matching pursuit (OMP), gain-phase error, auto-calibration

## Abstract

Radar coincidence imaging (RCI) is a high-resolution staring imaging technique without the limitation of relative motion between target and radar. The sparsity-driven approaches are commonly used in RCI, while the prior knowledge of imaging models needs to be known accurately. However, as one of the major model errors, the gain-phase error exists generally, and may cause inaccuracies of the model and defocus the image. In the present report, the sparse auto-calibration method is proposed to compensate the gain-phase error in RCI. The method can determine the gain-phase error as part of the imaging process. It uses an iterative algorithm, which cycles through steps of target reconstruction and gain-phase error estimation, where orthogonal matching pursuit (OMP) and Newton’s method are used, respectively. Simulation results show that the proposed method can improve the imaging quality significantly and estimate the gain-phase error accurately.

## 1. Introduction

Radar coincidence imaging (RCI), originated from the classical coincidence imaging in optical systems, is a novel staring imaging technique [1,2,3]. The RCI can realize high-resolution imaging without the limit of the target relative motion, and operate under the observing geometry of forward-looking/staring, with significant potentials for resolution enhancement, interference and jamming suppression. In RCI, the time-space independent and stochastic waveforms are transmitted, thus the spatial variety of wavefront is increased. The scatterers within a beam then reflect different signals according to their respective locations, so the super-resolution within a beam emerges, compared with other imaging techniques.

In RCI, sparse recovery is commonly used as the scatterers of targets are often distributed sparsely in some radar imaging applications. RCI can then be modeled as a linear inverse problem with a sparsity constraint in sparsity-driven approaches. Solving the problem depends on the perfect prior knowledge of the system. However, gain-phase errors among the transmitter-receiver pairs exists generally in RCI, which results in the dictionary mismatch and induces the performance to degrade significantly, since the imaging performance highly depends on presetting an appropriate sparsifying dictionary based on an accurate prior known model.

Various studies have been presented on gain-phase errors, most of which are based on eigenstructure and concentrate on angle estimation in sensor array. In [4], a method for simultaneously estimating direction-of-arrival (DOA) and gain-phase error without the joint iteration is proposed. In [5], a method based on eigendecomposition of the Hadamard product of the covariance matrix and its conjugate is proposed for DOA with gain-phase error. Algorithms for joint angles and array gain-phase error estimation in bistatic multiple-input multiple-output (MIMO) radar based on reduced-dimension multiple signal classification (MUSIC) and based on trilinear decomposition are proposed in [6,7,8]. In [9], an estimation of signal parameters via rotational invariance techniques (ESPRIT)-based method is presented to estimate the gain-phase errors of both transmission and reception arrays in bistatic MIMO radars. Similarly, an ESPRIT-like algorithm is proposed to realize angle estimation without any information of the gain and phase uncertainties [10]. In [11], two new estimation algorithms are proposed to estimate the gain and phase errors, *i.e.*, estimation algorithm for the conventional data model (EACDM) and estimation algorithm for the improved data model (EAIDM).

These methods are less sensitive to phase error [12] but lack adaptation to demanding scenarios with low signal-to-noise ratio (SNR), limited snapshots and spatially adjacent sources, just as their counterparts do in accurately calibrated arrays. Exploiting the sparseness previously, an adaptive sparse representation algorithm is proposed to improve the performance of source localization with respect to the gain/phase errors by dynamically calibrating the overcomplete basis and adaptively estimating the sparse solution [13]. Furthermore, from the Bayesian statistics perspective, a unified framework based on sparse Bayesian learning is formulated to realize array calibration and source DOA estimation, and a sparse Bayesian array calibration (SBAC) method is then proposed in [12]. Using variational Bayesian inference, an array auto-calibration sparse Bayesian learning (AASBL) algorithm in the full conjugate Bayesian framework is proposed to achieve DOA estimation with gain/phase errors in [14].

In the present report, we focus on the gain-phase error calibration in sparsity-driven RCI. Inspired by the sparsity-driven iterative method for joint synthetic aperture radar (SAR) imaging and phase error correction proposed in [15], we propose a sparse auto-calibration method for joint imaging and gain-phase error calibration on the sparse recovery framework. The method involves an iterative algorithm, each iteration of which consists of consecutive steps of target reconstruction and gain-phase error estimation, where orthogonal matching pursuit (OMP) and Newton’s method are adopted, respectively. The proposed method can exactly reveal the behavior of the gain-phase errors without any approximations being required. Numerical simulations show that the method realizes the imaging robustly and achieves both high resolution and outstanding imaging quality in the presence of gain-phase error, furthermore, its implementation is simple and fast without changing the algorithm parameters.

The rest of the report is organized as follows. In Section 2, the RCI model with gain-phase errors in the range-azimuth space is presented. Section 3 presents the sparse auto-calibration RCI method in detail. In Section 4, the performance of the proposed method is verified by numerical examples. Finally, Section 5 concludes the report.

## 2. RCI Model with Gain-Phase Errors

The RCI can be realized by a multitransmitter configuration to transmit time-independent and group-orthogonal waveforms [1]. Then, a monostatic radar with M transmitters and one receiver is considered in the present report, each transmitter emits an independent stochastic waveform. Thus, the echo component of each scattering center can be extracted and then correlated to their respective positions to obtain the spatial distribution of scattering centers.

**Figure 1:**
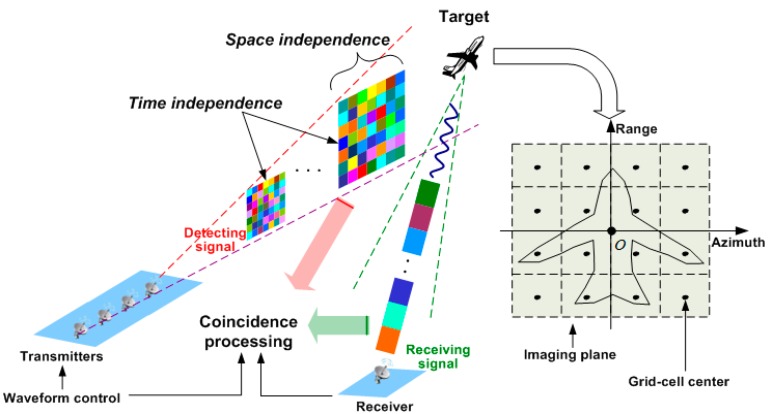
RCI Geometry.

The RCI geometry is illustrated in Figure 1. The imaging plane is a range-azimuth space. In sparsity-driven RCI, the continuous imaging plane is discretized to generate U azimuth cells, V range cells and associated cell size ${\text{Δ}}_{\theta }$, ${\text{Δ}}_{R}$. Thus the grid-cell number is K=UV. Denoted by $\beta_{k}$ the scattering coefficient of the scattering center exactly located at the pre-discretized k-th grid-cell center, *i.e.*, $r_{k}=\left(\theta_{k},R_{k}\right)$, and $\beta_{k}=0$ for the grid-cell without scattering center.

As the backscattering of a radar target in the high-frequency region can be approximated as coming from a few dominant scattering centers [16], the target is assumed to be composed of a very limited amount of strong scattering centers. Then, the number of scattering centers is much smaller than that of grid-cells in the image plane, which means the RCI image is spatially sparse.

The echo is a linear combination of all the scatterers’ reflected waveforms from all the transmitters. Considering the phase error, the echo at the receiver can be expressed as

(1) $$y\left(t\right)=\sum_{k\in S}\sum_{m=1}^{M}\beta_{k}a_{m}e^{j\varphi_{m}}St_{m}\left(t-\tau_{m}^{k}\right)+w\left(t\right)$$

where $St_{m}\left(t\right)$ is the signal emitted by the m-th transmitter, $a_{m}$ and $\varphi_{m}$ are the gain and phase errors between the m-th transmitter and the receiver pair, respectively. w(t), an independent complex Gaussian random process, denotes the noise at the receiver. $\tau_{m}^{k}$ is the propagation delay corresponding to the m-th transmitter and receiver with respect to the k-th scatterer. In addition, the RCI formula needs a detecting signal [1], which is simply structured as

(2) $$S\left(t,r_{k}\right)=\sum_{m=1}^{M}a_{m}e^{j\varphi_{m}}St_{m}\left(t-\tau_{m}^{k}\right)$$

Thus, the echo can be expressed as the superposition of the detecting signals, *i.e.*, $y\left(t\right)=\sum_{k\in S}\beta_{k}S\left(t,r_{k}\right)$. After sampling the echo, the imaging equation can be given as follows

(3) $$\begin{array}{l} \;y=S\cdot \beta +w\\ \left[\begin{array}{c} y\left(t_{1}\right)\\ y\left(t_{2}\right)\\ \vdots \\ y\left(t_{N}\right) \end{array}\right]=\left[\begin{array}{cccc} S\left(t_{1},r_{1}\right) & S\left(t_{1},r_{2}\right) & \cdots & S\left(t_{1},r_{K}\right)\\ S\left(t_{2},r_{1}\right) & S\left(t_{2},r_{2}\right) & \cdots & S\left(t_{2},r_{K}\right)\\ \vdots & \vdots & \cdots & \vdots \\ S\left(t_{N},r_{1}\right) & S\left(t_{N},r_{2}\right) & \cdots & S\left(t_{N},r_{K}\right) \end{array}\right]\cdot \left[\begin{array}{c} \beta_{1}\\ \beta_{2}\\ \vdots \\ \beta_{K} \end{array}\right]+\left[\begin{array}{c} w\left(t_{1}\right)\\ w\left(t_{2}\right)\\ \vdots \\ w\left(t_{N}\right) \end{array}\right] \end{array}$$

where N is the number of samples, S is the dictionary in sparse recovery framework, y, w and β are the echo, noise and unknown scattering coefficient vector, respectively. Thus, the imaging model reduces to a familiar linear model used in most applications of sparse recovery.

However, since the gain-phase error cannot be known accurately in practice, then S can be rewritten as S(a,φ) involving the gain error a and phase error φ, where $a={\left[a_{1},\cdots ,a_{M}\right]}^{T}$ and $\varphi ={\left[\varphi_{1},\cdots ,\varphi_{M}\right]}^{T}$. Then, Equation (3) can be rewritten as

(4) y=S(a,φ)·β+w

As a and φ are generally unknown, the true dictionary S(a,φ) is unknown and then β could not be reconstructed directly based on the conventional sparse recovery algorithms. Therefore, a sparse auto-calibration method, which is presented in the following section, is proposed to solve the problem.

## 3. Sparse Auto-Calibration RCI Method

Conventional sparsity-driven radar imaging methods assume that the model thus contains no errors, and the dictionary S(a,φ) is precisely known. The generally existing gain-phase errors would destroy the structure of the dictionary and lead to the direct use of sparse recovery methods failing. Then, for RCI with gain-phase errors, besides the target reconstruction, the gain-phase errors also need to be estimated. In the present report, a nonquadratic regularization-based method is proposed to solve the problem of joint target reconstruction and error estimation with the following cost function [15]

(5) $$J\left(\beta ,a,\varphi \right)={\left\|y-S\left(a,\varphi \right)\beta \right\|}_{2}^{2}+\lambda {\left\|\beta \right\|}_{1}$$

where λ is the regularization parameter, which specifies the strength of the contribution of the target regularization term into the solution.

The target and the gain-phase error can be obtained as

(6) $$\left[\beta ,a,\varphi \right]=\underset{\beta ,a,\varphi }{\arg\min}J\left(\beta ,a,\varphi \right)$$

By solving Equation (6), an alternating iterative minimization method is presented to realize the auto-calibration RCI with gain-phase error, based on the sparse recovery framework. The proposed method works by jointly reconstructing the target and estimating the gain-phase error. In the first step of each iteration, the cost function is minimized with respect to the target and the target is reconstructed for given gain-phase error. In the second step, the gain and phase errors are then estimated separately, using the target reconstruction results. Then, the estimated gain-phase error is used to update the dictionary S(a,φ), and the method passes to the next iteration.

In addition, we terminate the method if ${\left\|\beta^{i+1}-\beta^{i}\right\|}_{2}^{2}/{\left\|\beta^{i}\right\|}_{2}^{2}<\eta$ or the maximum number of iterations $I_{\max}$ is reached, where η is a predetermined threshold and the superscript i refers to the iteration.

Based on the discussions above, the procedure of the method flow is outlined in Algorithm 1. **Algorithm 1** Sparse auto-calibration radar coincidence imaging (RCI) method**Input**: y, S(a=1,φ=0), $I_{\max}$, η**Initialization**: i=0, a=1, φ=0**Iteration**: While continuing, if not converged, do  **Step 1**: Target reconstruction $\beta^{i+1}=\underset{\beta }{\arg\min}J\left(\beta ,a^{i},\varphi^{i}\right)$  **Step 2**: Gain error estimation Phase error estimation $a^{i+1}=\underset{\varphi }{\arg\min}J\left(\beta^{i+1},a,\varphi^{i}\right)$     Phase error estimation $\varphi^{i+1}=\underset{\varphi }{\arg\min}J\left(\beta^{i+1},a^{i\text{+}1},\varphi \right)$  **Step 3**: Gain-phase error compensation, update $S\left(a^{i+1},\varphi^{i+1}\right)$  **Step 4**: Let i=i+1 and check for convergence: ${\left\|\beta^{i+1}-\beta^{i}\right\|}_{2}^{2}/{\left\|\beta^{i}\right\|}_{2}^{2}<\eta$ or $i=I_{\text{max}}$ end while **Output**: Reconstructed scattering coefficient vector

In the aforementioned method, Steps 1 and 2 are the major steps of the method. Thus, we provide the details of Steps 1 and 2.

### 3.1. Target Reconstruction

In Step 1, the target is reconstructed when the gain-phase error is given. It can be denoted as:

(7) $$\beta^{i+1}=\underset{\beta }{\arg\min}\left\{{\left\|y-S\left(a^{i},\varphi^{i}\right)\beta \right\|}_{2}^{2}+\lambda {\left\|\beta \right\|}_{1}\right\}$$

It can be seen that Equation (7) is a standard form of compressive sensing-based or sparsity-driven imaging formula. This problem can be solved by some existing methods, such as convex relaxation methods (e.g., basis pursuit de-noising), greedy iterative methods (e.g., OMP), and non-convex minimization methods (e.g., sparse Bayesian learning). In our proposed method, OMP is used to reconstruct the target by conducting a greedy strategy that iteratively selects the basis vector, for its advantages of unrequirement of prior knowledge, low computational burden and implementation complexity.

### 3.2. Gain-Phase Error Estimation

The gain-phase errors (a,φ) should be considered as unknown deterministic parameters as they are not varying generally during the entire coherent processing interval. In Step 2, the gain and phase errors are estimated in an alternating manner. The gain error is estimated as

(8) $$a^{i+1}=\underset{a}{\arg\min}\left\{{\left\|y-S\left(a,\varphi^{i}\right)\beta^{i+1}\right\|}_{2}^{2}+\lambda {\left\|\beta^{i+1}\right\|}_{1}\right\}$$

Since $\lambda {\left\|\beta^{i+1}\right\|}_{1}$ is a constant, Equation (8) can be rewritten as

(9) $$a^{i+1}=\underset{a}{\arg\min}\left\{{\left\|y-S\left(a,\varphi^{i}\right)\beta^{i+1}\right\|}_{2}^{2}\right\}$$

Define $f\left(a,\varphi \right)={\left\|y-S\left(a,\varphi \right)\beta^{i+1}\right\|}_{2}^{2}$ as the objective function. Clearly, Equation (9) is a nonlinear least-squares problem, which is not tractable to obtain the closed-form expression for updating $a^{i+1}$. We instead use Newton’s method [17] to solve the problem, which proceeds in a direction of descent to locate the minimum after a number of iterations and reveals the behavior of gain error with no approximation being required. Denoting by $a^{i}$ the parameter estimation at the i-th iteration, the updated $a^{i+1}$ estimate is then computed as

(10) $$a^{i+1}=a^{i}-{\left[\nabla_{a}^{2}f\left(a^{i},\varphi^{i}\right)\right]}^{-1}\left[\nabla_{a}f\left(a^{i},\varphi^{i}\right)\right]$$

where $\nabla_{a}f\left(a^{i},\varphi^{i}\right)$ and $\nabla_{a}^{2}f\left(a^{i},\varphi^{i}\right)$ represent the gradient and Hessian with respect to the gain error, respectively. After derivation and simplification, we have

(11) ∇af(ai,φi)=−2Re((B(ai,φi))Hw⌢)

(12) $$\nabla_{a}^{2}f\left(a^{i},\varphi^{i}\right)=2\text{Re}\left({\left(B\left(a^{i},\varphi^{i}\right)\right)}^{H}B\left(a^{i},\varphi^{i}\right)\right)$$

(13) $$\overset{⌢}{w}=y-S\left(a^{i},\varphi^{i}\right)\beta^{i+1}$$

(14) $$B\left(a^{i},\varphi^{i}\right)=\left[b_{1}\left(a^{i},\varphi^{i}\right),\cdots ,b_{M}\left(a^{i},\varphi^{i}\right)\right]$$

where Re(·) denotes the real part, $b_{m}\left(a^{i},\varphi^{i}\right)=\overset{⌢}{S}t_{m}^{a}\beta^{i+1}$, $\overset{⌢}{S}t_{m}^{a}=\left[\overset{⌢}{S}t_{m}^{a}\left(r_{1}\right),\cdots ,\overset{⌢}{S}t_{m}^{a}\left(r_{K}\right)\right]$, $\overset{⌢}{S}t_{m}^{a}\left(r_{k}\right)=e^{j\varphi_{m}^{i}}{\left[St_{m}\left(t_{1}-\tau_{m}^{k}\right),\cdots ,St_{m}\left(t_{N}-\tau_{m}^{k}\right)\right]}^{T}$.

The phase error is estimated as

(15) $$\varphi^{i+1}=\underset{\varphi }{\arg\min}\left\{{\left\|y-S\left(a^{i\text{+}1},\varphi \right)\beta^{i+1}\right\|}_{2}^{2}+\lambda {\left\|\beta^{i+1}\right\|}_{1}\right\}$$

Using the same way as the updated $a^{i+1}$, the updated $\varphi^{i+1}$ estimate is then computed as

(16) $$\varphi^{i+1}=\varphi^{i}-{\left[\nabla_{\varphi }^{2}f\left(a^{i\text{+}1},\varphi^{i}\right)\right]}^{-1}\left[\nabla_{\varphi }f\left(a^{i\text{+}1},\varphi^{i}\right)\right]$$

(17) ∇φf(ai+1,φi)=−2Im((D(ai+1,φi))Hw⌢)

(18) ∇φ2f(ai+1,φi)=2diag(Re((D(ai+1,φi))Hw⌢))+2Re((D(ai+1,φi))HD(ai+1,φi))

(19) $$D\left(a^{i\text{+}1},\varphi^{i}\right)=\left[d_{1}\left(a^{i\text{+}1},\varphi^{i}\right),\cdots ,d_{M}\left(a^{i\text{+}1},\varphi^{i}\right)\right]$$

where diag(·) is the diagonalization operation, Im(·) denotes the imaginary part. Like the definition of $b_{m}\left(a^{i},\varphi^{i}\right)$, $d_{m}\left(a^{i\text{+}1},\varphi^{i}\right)$ is defined a $d_{m}\left(a^{i\text{+}1},\varphi^{i}\right)=\overset{⌢}{S}t_{m}^{\varphi }\beta^{i+1}$, $\overset{⌢}{S}t_{m}^{\varphi }=\left[\overset{⌢}{S}t_{m}^{\varphi }\left(r_{1}\right),\cdots ,\overset{⌢}{S}t_{m}^{\varphi }\left(r_{K}\right)\right]$, $\overset{⌢}{S}t_{m}^{\varphi }\left(r_{k}\right)=a_{m}^{i+1}e^{j\varphi_{m}^{i}}{\left[St_{m}\left(t_{1}-\tau_{m}^{k}\right),\cdots ,St_{m}\left(t_{N}-\tau_{m}^{k}\right)\right]}^{T}$.

### 3.3. Discussions

In this part, more discussions are made to provide further insight into the proposed sparse auto-calibration RCI method.

In fact, from the Bayesian perspective, solving Equation (6) can be regarded as a maximum *a posteriori* (MAP) estimation [18]. The noise is assumed as a complex Gaussian random process, thus the likelihood model can be written as

(20) $$p\left(y|\sigma^{2}\right)={\left(\frac{1}{2\pi \sigma^{2}}\right)}^{N}\exp\left(-\frac{1}{2\sigma^{2}}{\left\|y-S\left(a,\varphi \right)\beta \right\|}_{2}^{2}\right)$$

where $\sigma^{2}$ denotes the noise variance. Taking the sparse prior into consideration, we assign β a widely used Laplace prior to induce sparsity.

(21) $$p\left(\beta |\varepsilon \right)={\left(\frac{\varepsilon }{2}\right)}^{K}\exp\left(-\varepsilon \sum_{k=1}^{K}\left|\beta_{k}\right|\right)$$

where ε is the scale parameter of Laplace distribution. In the Bayesian framework, we have

(22) $$p\left(\beta |y,\sigma^{2},\varepsilon \right)=\frac{p\left(y|\sigma^{2},\beta \right)\cdot p\left(\beta |\varepsilon \right)}{p\left(y|\sigma^{2},\varepsilon \right)}$$

where the normalized factor $p\left(y|\sigma^{2},\varepsilon \right)$ is defined as $p\left(y|\sigma^{2},\varepsilon \right)\text{=}\int p\left(y|\sigma^{2},\beta \right)\cdot p\left(\beta |\varepsilon \right)d\beta$. Then, the MAP estimator is given by

(23) $$\overset{⌢}{\beta }=\underset{\beta \in {\mathbb{C}}^{K}}{\arg\max}\log\left(p\left(y|\sigma^{2},\beta \right)\cdot p\left(\beta |\varepsilon \right)\right)$$

Substitute Equations (20) and (21) into Equation (23), then we have

(24) $$\begin{array}{l} \overset{⌢}{\beta }=\underset{\beta \in {\mathbb{C}}^{K}}{\arg\min}\left(\frac{1}{2\sigma^{2}}{\left\|y-S\left(a,\varphi \right)\beta \right\|}_{2}^{2}+\varepsilon {\left\|\beta \right\|}_{1}\right)\\ \;=\underset{\beta \in {\mathbb{C}}^{K}}{\arg\min}\left({\left\|y-S\left(a,\varphi \right)\beta \right\|}_{2}^{2}+\lambda {\left\|\beta \right\|}_{1}\right) \end{array}$$

where $\lambda \text{=}2\varepsilon \sigma^{2}$ is the regularization parameter. Then, the MAP estimator can be realized by solving the optimization problem described in Equation (24) which is the same as Equation (6).

It is shown in Equation (24) that the regularization parameter λ is proportional to the noise power. In the regularization-based algorithm, the parameter should be estimated, which is difficult in practical applications, as both the noise variance and scale parameter cannot be accessed easily. In the present report, OMP is used to reconstruct the target. Hence, Problem (7) is solved without the estimation of the regularization parameter.

Next, we show the convergence of the proposed method. For mathematical convenience, we define the sequence $J_{i}=J\left(\beta^{i},a^{i},\varphi^{i}\right)$, which is the cost function value of the i-th iteration. As described in Equation (6), the alternating iterative method minimizes the cost function J(β,a,φ). Thus

(25) $$\beta^{i+1}=\underset{\beta }{\arg\min}J\left(\beta ,a^{i},\varphi^{i}\right)$$

(26) $$a^{i+1}=\underset{\varphi }{\arg\min}J\left(\beta^{i+1},a,\varphi^{i}\right)$$

(27) $$\varphi^{i+1}=\underset{\varphi }{\arg\min}J\left(\beta^{i+1},a^{i\text{+}1},\varphi \right)$$

Then, we can deduce that

(28) $$J\left(\beta^{i+1},a^{i},\varphi^{i}\right)\leq J\left(\beta^{i},a^{i},\varphi^{i}\right),\forall i$$

(29) $$J\left(\beta^{i+1},a^{i+1},\varphi^{i}\right)\leq J\left(\beta^{i+1},a^{i},\varphi^{i}\right),\forall i$$

(30) $$J\left(\beta^{i+1},a^{i\text{+}1},\varphi^{i+1}\right)\leq J\left(\beta^{i+1},a^{i\text{+}1},\varphi^{i}\right),\forall i$$

From Equations (28)–(30), the difference $J_{i\text{+1}}-J_{i}$ is deduced as

(31) $$\begin{array}{l} J_{i\text{+1}}-J_{i}\text{=}\left[J\left(\beta^{i+1},a^{i\text{+}1},\varphi^{i+1}\right)-J\left(\beta^{i+1},a^{i\text{+}1},\varphi^{i}\right)\right]\\ \text{ +}\left[J\left(\beta^{i+1},a^{i+1},\varphi^{i}\right)-J\left(\beta^{i+1},a^{i},\varphi^{i}\right)\right]\\ \text{ +}\left[J\left(\beta^{i+1},a^{i},\varphi^{i}\right)-J\left(\beta^{i},a^{i},\varphi^{i}\right)\right]\\ \;\leq \text{0} \end{array}$$

As shown in Equation (31), the sequence $J_{i}$ is decreasing and converges. Furthermore, the proposed method consists of two types of iterative procedure. For the target reconstruction step, the conventional OMP is used. The OMP algorithm is an iterative process and its convergence is analyzed in many literatures. For the gain-phase error estimation step, it is an unconstrained optimization problem which is solved by the Newton’s method whose convergence is guaranteed by the property of Newton method [19]. Consequently, the method is convergent in terms of the cost function.

In general, the proposed method may converge to a local or global minimum, which is connected with the choice of the starting point. Then, we initialize the gain-phase error with a=1 and φ=0, which means that the initial gain-phase error is zero. In addition, β should be initialized before the iterations. In the present report, the conventional OMP is conducted to obtain the initialization of β.

## 4. Numerical Simulations

In this section, simulations are carried out to verify the proposed sparse auto-calibration RCI method. An X-band RCI radar system with carrier frequency of 10GHz is considered. The transmitters are configured as a uniform linear array with M=8 and inter-element spacing d=0.5 m. The transmitters emit independent frequency-hopping waveforms with the bandwidth of 500 MHz. A range-azimuth imaging plane, covering 8 m × 0.08 rad, is discretized to 40 × 40 grid-cells. The gain and phase errors are randomly varying at [0.7, 1.3] and [−45°,45°], respectively. We initialize a=1, φ=0, $I_{\max}\text{=}200$ and *η* = 10^−3^. For the scattering coefficient vector β, we use the conventional OMP as our initialization of the proposed method.

### 4.1. Illustrative Example

**Figure 2:**
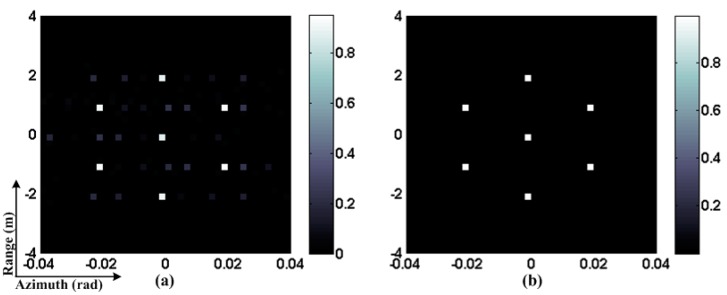
RCI results. (**a**) OMP; (**b**) Sparse auto-calibration method.

To illuminate the validity of the proposed method, we conduct a numerical simulation where the OMP algorithm is implemented as a comparison. Further, there are supposed to be seven ideal point scatterers in the imaging plane. Figure 2 shows the RCI results. Figure 2a is for the OMP algorithm. It can be seen that the image is defocused, many spurious scatterers exist, and the signal energy spills over the imaging plane because of the gain-phase error. Figure 2b is for our proposed method, where the target is reconstructed accurately due to the gain-phase error compensation. Therefore, the proposed method exhibits significant performance improvement.

**Figure 3:**
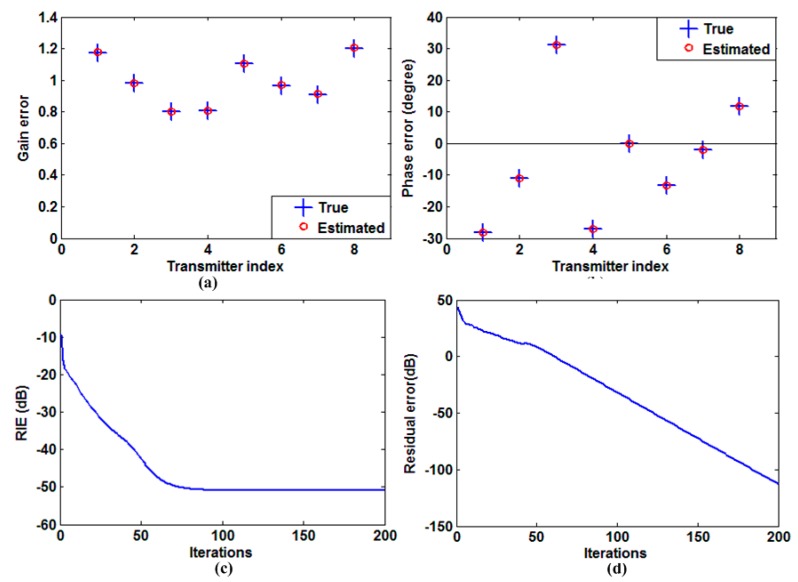
Auto-calibration performance. (**a**) Estimated and true gain error; (**b**) Estimated and true phase error; (**c**) RIE *versus* the number of iterations; (**d**) Residual error *versus* the number of iterations.

Then, we show the auto-calibration performance in Figure 3. It is clearly shown that both the gain and phase errors are estimated accurately. Figure 3c,d show the relative imaging error (RIE) and residual error with respect to the number of iterations. The RIE is defined as $20\log_{10}{\left\|\overset{⌢}{\beta }-\beta_{0}\right\|}_{2}^{2}/{\left\|\beta_{0}\right\|}_{2}^{2}$, where $\overset{⌢}{\beta }$ and $\beta_{0}$ denote the reconstructed and true value of β, respectively. Residual error is defined as $\left\|y-S\left(a,\varphi \right)\overset{⌢}{\beta }\right\|$, which is the model error after target reconstruction and error compensation. As shown in Figure 3c,d, the RIE and residual error reduce rapidly to a small value and change slightly after about 60 iterations, which means that the target is reconstructed perfectly and the model error is compensated accurately.

### 4.2. Performance under Different SNRs

**Figure 4:**
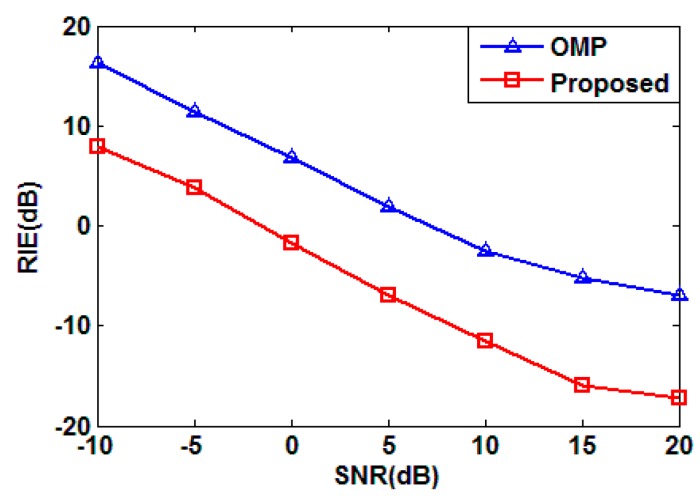
RIE *versus* SNR.

Note that the above simulation is conducted without noise. Then, we test RIEs under different SNRs, for the proposed method and conventional OMP algorithm, the result is shown in Figure 4. As shown in the figure, the imaging quality is improved significantly as the SNR increases, which means the proposed method is sensitive to noise. While compared with OMP, the proposed method improves the imaging performance by more than 8 dB from the RIE perspective.

**Figure 5:**
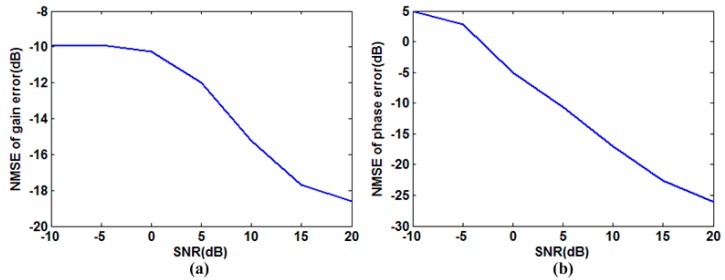
Gain-phase error estimation performance for various SNRs. (**a**) NMSE for gain error estimation *versus* SNR; (**b**) NMSE for phase error estimation *versus* SNR.

For the proposed sparse auto-calibration RCI method, both the target reconstruction and gain-phase error estimation are sensitive to noise. It is shown in Figure 4 that the performance of target reconstruction, which uses an OMP algorithm, degrades considerably when the noise power increases. Meanwhile, we show the normalized mean square error (NMSE) for gain-phase error estimation under different SNRs in Figure 5, and conclude that decreasing SNR would impair the gain-phase error estimation performance dramatically. Hence, the gain-phase error would not be compensated perfectly in the presence of noise, which worsens the reconstruction performance. Thus, the ways of increasing the SNR should be implemented in practical applications, for example, improving the power of the transmitting signal.

### 4.3. Performance under Different Target Scenes

**Figure 6:**
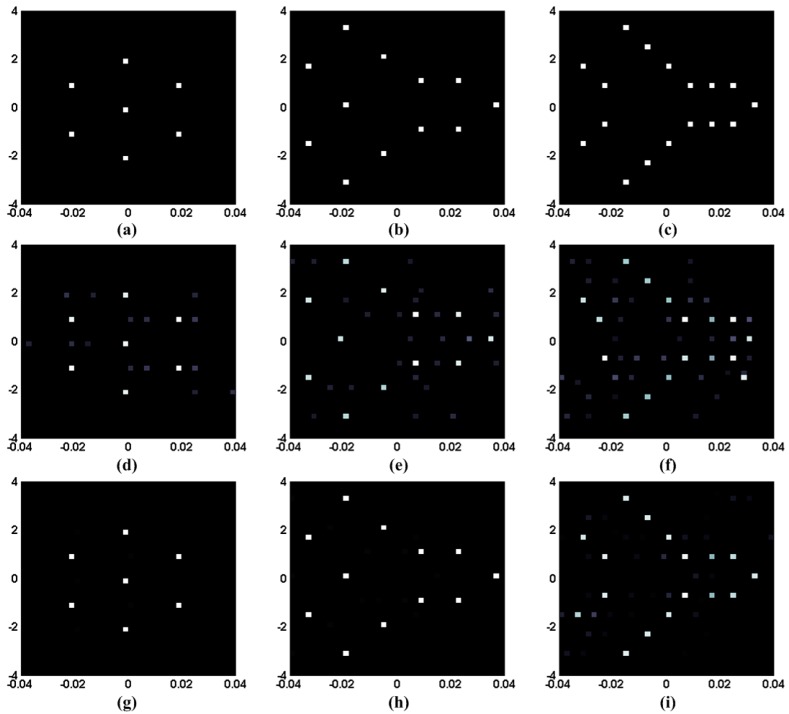
RCI results for different target scenes. (**a**–**c**) Three different target scenes; (**d**–**f**) Imaging results of OMP for the three target scenes; (**g**–**i**) Imaging results of the proposed method for the three target scenes.

The proposed sparse auto-calibration method is based on the assumption that the target is sparse, which means that the scatterers are widely separated and fewer than the grid-cells. Thus, the reconstruction performance may be affected by the target, more precisely, the sparsity of target. In this part, the numerical simulations are designed to test the performance under different target scenes which are shown in Figure 6a–c.

It can be concluded from Figure 6 that the proposed method achieves much sparser and focused images, making it of much practical significance in improving the image quality. Comparing with the results obtained by OMP, the spurious scatterers in the bottom three images are much less, and the three targets are identified clearly. However, the images become blurred as the complexity of targets increases. On the one hand, the less sparse target would make the target reconstruction more difficult, as it is based on the prior knowledge of sparsity. On the other hand, the gain-error estimation performance is also affected, since this iteration lies on the perfect reconstructed scattering coefficient vector as shown in Equations (11)–(14) and (16)–(19).

## 5. Conclusions

This report has proposed a sparse auto-calibration method to realize the gain-phase error calibration in sparsity-driven RCI. The proposed method can jointly reconstruct the target and estimate the gain-phase error. It uses an alternating iterative algorithm, which cycles through steps of target reconstruction and gain-phase error estimation and compensation. For the two steps, OMP algorithm and Newton’s method are used, respectively. The proposed method can estimate the gain-phase error accurately and improve the reconstruction performance significantly. Numerical experiments have been presented to show the effectiveness and outstanding imaging performance of the method, which shows the potential for the method to be applied in a practical RCI system. Although only the case of RCI is considered, the proposed method in the present report can be extended to other imaging radar systems, such as generalized MIMO radar imaging and passive radar imaging.

However, in the presented report, Problem (7) is solved by OMP without the estimation of the regularization parameter. To estimate the regularization parameter is difficult in practical applications but can be performed in the sparse Bayesian learning (SBL) framework. When SBL is introduced, the individual Gaussian prior is assigned to β, then $p\left(y|\sigma^{2},\varepsilon \right)$ shown in Equation (22) can be derived in closed form, and the Bayesian analysis can be completed. Involving Bayesian analysis, all the necessary parameters can be estimated. For the classical Newton’s method, calculating the Hessian numerically involves a large amount of computation, and it is not easy to prove the invertibility of Hessian. To solve the problems, the quasi-Newton method can be used where the Hessian and its inverse matrix can be approximated using an appropriate updating technique. Moreover, the scatterers are assumed be sparse and widely separated to guarantee the imaging, which limits the resolution. These problems are open issues that are beyond the scope of this report and will be investigated deeply in our future work.
